# Evaluating Complex Interventions Using Qualitative Longitudinal Research: A Case Study of Understanding Pathways to Violence Prevention

**DOI:** 10.1177/10497323211002146

**Published:** 2021-05-13

**Authors:** Shelly Makleff, Jovita Garduño, Rosa Icela Zavala, Jimena Valades, Florencia Barindelli, Mariana Cruz, Cicely Marston

**Affiliations:** 1 London School of Hygiene and Tropical Medicine, London, United Kingdom; 2 Monash University, Melbourne, Victoria, Australia; 3 Fundación Mexicana para la Planeación Familiar, A.C., Mexico City, Mexico; 4 Independent, New York; 5 Independent, Montevideo, Uruguay; 6 IPPFWHR Mexico, Mexico City, Mexico

**Keywords:** intimate partner violence, young people, prevention, qualitative, repeat interviews, qualitative longitudinal research, Mexico

## Abstract

Evaluating social change programs requires methods that account for changes in context, implementation, and participant experience. We present a case study of a school-based partner violence prevention program with young people, where we conducted 33 repeat interviews with nine participants during and after an intervention and analyzed participant trajectories. We show how repeat interviews conducted during and after a social change program were useful in helping us understand how the intervention worked by providing rich contextual information, elucidating gradual shifts among participants, and identifying aspects of the intervention that appear to influence change. Long-term effects of social change interventions are very hard to quantify or measure directly. We argue that a qualitative longitudinal approach provides a way to measure subtle changes that can serve as proxies for longer term impacts.

## Introduction

Evaluating social change programs requires methods that account for changes in context, implementation, and participant experience. Social change programs are complex: They are nonlinear, often implemented in nonstandardized ways, comprise multiple program components that interact, and manifest differently depending on context (Petticrew, 2011). Complex interventions “may change with time, for pragmatic reasons, change of external factors, or as a result of learning during the evaluation” (Craig et al., 2008, p. 14). Evaluations would benefit from taking into consideration the different types of temporal change that influence complex interventions, such as those focusing on social change. Yet, there is little concrete guidance on how to do so (Craig et al., 2008; Moore et al., 2015), and evaluations often use “linear, measurable, and causative” tools—even though these approaches may not be applicable to social change processes (Lacayo et al., 2008, p. 128).

There is growing consensus that evaluations of complex interventions—such as social change programs—would benefit from theorizing how interventions work and understanding which components contribute to change, for whom, and in what situations and contexts (Astbury & Leeuw, 2010; Bonell et al., 2012; Howarth et al., 2016; Moore et al., 2015). Theory of change, which can be defined as an articulation of or set of hypotheses about why and how a program will work (Weiss, 1995), can bring a theory-based approach to the evaluation of complex interventions (Silva et al., 2014). The theory of change approach has been used in different contexts to explore social change and why it happens (James, 2011; Retolaza Eguren, 2011). There has been an increasing focus on developing culturally and contextually responsive theories of change of social change programs, particularly for gender-based and intimate partner violence (IPV) prevention, as a way to explore the pathways to prevention (Eisenbruch, 2018; Fulu & Kerr-Wilson, 2015; Jewkes et al., 2015; Michau et al., 2015; Moosa et al., 2012).

Qualitative longitudinal methods, which entail collecting qualitative data over time to center inquiry on temporality and change (Corden & Millar, 2007), can be used to examine how and why change happens in relation to sociocultural context (Holland et al., 2006). This makes them well suited for evaluating social policies or programs in their real-life settings (Calman et al., 2013; Lewis, 2007; Thomson, 2007; Thomson & McLeod, 2015). Because social change programs such as IPV prevention interventions do not always achieve measurable community-wide transformation, evaluations should also be designed to learn about the sometimes small and incremental shifts in attitudes or behaviors that can still occur (Gibbs et al., 2019; McLean et al., 2019); qualitative longitudinal methods may help detect such shifts. In this article, we consider the use of repeat interviews—a qualitative longitudinal data collection method—as an evaluation strategy to take temporal change into consideration and examine pathways to social change. We do so using the case of a school-based evaluation of an IPV prevention program with young people in Mexico City.

Recent evaluations of IPV prevention interventions in Mozambique, Nepal, Rwanda, and South Africa have included repeat interviews as part of larger evaluations to better understand how an intervention may contribute to change or to learn about implementation processes (Burke et al., 2019; Hatcher et al., 2020; McGhee et al., 2019; Stern & Heise, 2019). These studies varied in interview frequency and timing: The study in Nepal conducted two interviews 4 months apart (McGhee et al., 2019), whereas the other three waited 1 year between interviews. The Rwanda study conducted the first interview before the intervention (Stern & Heise, 2019); the rest started interviews during the intervention (Hatcher et al., 2020; McGhee et al., 2019) or after it ended (Burke et al., 2019). None of the studies gathered qualitative data at shorter intervals during the intervention, potentially limiting their ability to detect gradual or subtle shifts that might be occurring on the nonlinear pathways to change.

Our analysis explores evaluation data gathered using repeat interviews conducted with young people in Mexico City during and after their participation in a weekly comprehensive sexuality education program, which aimed to prevent IPV and encourage nonviolent and equitable relationships. We examine three questions about using repeat interviews in evaluations of school-based interventions. First, in what ways can repeat interviews provide information about pathways to intervention impact? Second, are repeat interviews during and after an intervention feasible and acceptable to participants? Third, what are the benefits and challenges of using repeat interviews during and after an intervention as strategy for the evaluation of social change interventions?

## Method

### Description of the Intervention

The repeat interviews analyzed here come from an evaluation of a comprehensive sexuality education program conducted at one school in Mexico City in 2017 and 2018. The program had been recently redesigned by the Mexican nonprofit organization Fundación Mexicana para la Planeación Familiar, A.C. (Mexfam), to strengthen its IPV prevention content. The course was delivered by paid health educators below 30 years of age, to groups of approximately 20 secondary school students between the ages of 14 and 17. Sessions were to take place weekly in classrooms over one semester, in 10 two-hour sessions. Health educators implemented a curriculum that aimed to shift beliefs and promote critical reflection about gender, violence, and sexuality. The 20-hour curriculum did so through participatory sessions that addressed a range of sexual and reproductive health topics, including sexual diversity, sexually transmitted infections, and contraception. The final sessions of the course directly addressed healthy relationships and IPV. Gender was a cross-cutting theme throughout the curriculum, which took a gender-transformative approach, that is, aimed to shift harmful gender norms (Dworkin & Barker, 2019; Dworkin et al., 2015).

At the beginning of the project, the partner organizations jointly developed a theory of change to articulate our hypotheses about how the school-based intervention might prevent or reduce IPV. This theory of change, which drew from practice-based knowledge and academic literature, served as a theoretical framework for the study. Throughout data collection and analysis, we iteratively refined the theory of change and identified four pathways of change through which the intervention might contribute to IPV prevention: (a) communicating about relationships, sexuality, and violence; (b) taking protective and preventive actions to promote equitable and less violent relationships; (c) accessing violence-related and sexual and reproductive health services; and (d) shifting beliefs and behaviors related to gender, sexuality, and violence (Makleff, 2020). The evaluation was designed to explore these hypotheses and understand how and why the program worked in its particular context.

### Evaluation Design

The evaluation was coproduced by International Planned Parenthood Federation Western Hemisphere Region, the London School of Hygiene and Tropical Medicine (LSHTM), and Mexfam, and employed a longitudinal quasi-experimental design. Data collection methods with students included baseline and endline surveys, focus group discussions, endline interviews, and repeat interviews. We also conducted focus group discussions with teachers and health educators, and observed the intervention classroom sessions, recording observations using field notes. More details about the course activities, study setting, research design, data collection, and evaluation findings are published elsewhere (Makleff, Billowitz, et al., 2020; Makleff, Garduño, et al., 2020). This article focuses on the data gathered using repeat interviews, and draws on complementary information gathered using other data collection methods when necessary to provide context.

### Informed Consent

All students in the intervention classrooms (a total of 185 participants) were required to attend the comprehensive sexuality education course as part of their ongoing school activities. Parents and guardians could not withdraw students from this course, but were given the option to exclude students from the study-related data collection, specifically questionnaires, interviews, and focus group discussions. Eligible students (14–17 years old, who participated in the intervention and had parent or guardian consent) were invited to participate in self-administered baseline and endline questionnaires, one-time in-depth interviews, repeat interviews, or focus group discussions, completing a separate assent process for each data collection method.

### Recruitment

Overall, 124 intervention participants completed the baseline questionnaire, of whom 87 reported any past experience of sexual contact, romantic relationships, or IPV at baseline. We randomly selected students from these 87 participants to invite for repeat interviews. We sought 10 such participants, with equal distribution by gender, to ensure diverse experiences while maintaining a manageable quantity of data. Ten of the 15 young people we approached in the first 2 months of the study agreed to participate, with one eventually withdrawing, leaving nine repeat interview participants. Repeat interviews were one of a range of complementary data collection methods designed to build a multifaceted understanding of how the program influenced participants and their processes of change, and the number of participants was chosen to provide the benefits of longitudinal data while also taking into account issues of feasibility in generating and analyzing the large volumes of data per longitudinal participant.

### Ethics

This research addressed issues of violence and relationships among 14- to 17-year-olds. All research team members were trained on research ethics for working with vulnerable populations such as young people. We prioritized protection of participants and their well-being throughout the research process, following ethical guidelines for research of this nature (Devries et al., 2015; Jewkes et al., 2012). Participants received a gift card of 200 Mexican pesos (equivalent to US$10 or a 1-month cellular phone data plan) after each interview to compensate them for their time. We also offered participants free counseling and sexual and reproductive health services at Mexfam clinics and at a school-based health fair. We obtained ethical approval in Mexico (Bioética y Ciencia para la Investigación) and the United Kingdom (LSHTM Ethics Committee).

### Participants

Nine participants participated in the repeat interviews. Seven completed four interviews, one had three interviews, and one participated in two—a total of 33 interviews. The nine participants—five young women and four young men—were distributed between four different implementation groups of approximately 20 participants each. At baseline, two were 14 years old, five were 15, one was 16, and one was 17 years old. All nine reported ever having a relationship, six ever having sexual contact, three having experienced IPV, and two having experienced sexual contact against their will.

### Data Collection

Interviews took place every 1 to 2 months over a 6-month period, both during and after the intervention (timeline shown in Table 1). We had intended to conduct the first interviews with each participant before the start of the intervention, but this was not possible because of delays in recruitment followed by earthquake-related school closures. The first interview was conducted early in the intervention semester, before the sessions about IPV, to provide context about the participant’s personal circumstances that might influence their experience in the intervention. Questions focused on social and family contexts (e.g., how do you get along with your family), relationship and sexual experiences (e.g., what do you like most/least about your relationship), perceptions of IPV (e.g., what forms of dating violence do you know of), observations about their peers’ dating experiences (e.g., do you know of dating violence at your school), and intended behaviors (e.g., what would you do if you saw a couple fighting).

**Table 1.** Repeat Interview Timeline, by Participant.

Participant	Month 1	Month 2	Month 3	Month 4	Month 5	Month 6	Number of interviews
1							4
2							3
3							4
4							4
5							4
6							4
7							4
8							4
9							2
Number of interviews	2	8	6	8	0	9	33
Intervention^a^							

a In September 2017, the school was closed for nearly 3 weeks following a significant earthquake in Central Mexico, interrupting the intervention.

Shaded cells indicate months when the interviews and intervention took place.

In each subsequent interview, the interviewer asked follow-up questions about experiences with relationships and IPV that had been raised in the prior session, and asked about changes in their lives since the last interview. In addition, questions focused on communicating about sexuality and relationships (e.g., have you recently had conversations about sex), information seeking (e.g., since the course began, have you looked for information about sex or dating), recent sessions of the intervention and how they and their classmates responded to the activities (e.g., which parts do you agree with, how do you think other students received the information), the influence of the course (e.g., have you put anything you learned into practice), and the nature of discussions during the activities (e.g., what are the most interesting conversations). In the final interview, which took place approximately 2 months after the intervention ended, we added questions to explore participants’ experiences with the repeat interviews, as well as questions about the impact of the intervention on their peers (e.g., has anything changed in your group during the course, such as how the students talk, interact, what they discuss) and on themselves (e.g., thinking of your own experience in the course, has anything changed in you).

The interviewer was a Mexfam staff member, and interviews took place in a private space at the school or at Mexfam’s headquarters nearby. Interviews lasted between 30 minutes and 2 hours. After each interview, the interviewer wrote field notes about the experience. Interviews were audio recorded with permission and transcribed in original Spanish by professional transcriptionists and Mexfam staff members. All transcriptions were quality checked by the research team.

### Data Analysis

We reviewed the interview field notes and listened to the full audio of each interview as it became available, and then developed a list of follow-up questions to incorporate into the subsequent interview. These questions focused on understanding each participant’s experiences in the intervention over time, gaining clarity on narratives that were challenging to interpret, probing on any apparent inconsistencies between their responses at different times, and exploring themes we identified as salient across the body of interviews. In this respect, the data collection and analysis followed a “progressive focusing” approach, iteratively adjusting data collection and analysis processes as we became familiarized with the data and refined our focus of inquiry based on earlier findings (Schutt & Chambliss, 2013; Sinkovics & Alfoldi, 2012; Stake, 1981).

We reviewed interview transcripts and field notes to explore the ways in which repeat interviews may provide information about pathways to intervention impact. We focused this analysis on one theory of change pathway: taking protective and preventive actions to promote equitable and less violent relationships. We analyzed data from the 33 repeat interviews following the five steps of framework analysis (Ritchie & Spencer, 1994). The first step, familiarization with the data, entailed listening to interview audio and reading transcripts. Second, we developed a thematic framework to describe the data, using the outcomes and pathways from the theory of change. Third, indexing involved coding transcripts according to the categories drawn from the theoretical framework. Fourth, for charting, we used “time-ordered, sequential” matrices to summarize any change for each participant (Grossoehme & Lipstein, 2016) within the key themes identified in the theory of change, such as beliefs about relationships and violence. We first created a matrix for each participant, with one column per interview and one row per theme. We then created a consolidated matrix to summarize the change, or lack thereof, within each theme across all participants. Fifth, for the mapping and interpretation phase, we discussed these matrices as a team and wrote analytic memos to reflect on patterns, similarities, or differences between cases and contextual factors that appeared to influence the changes we identified within cases and across the sample.

We used iterative memo writing to draft an exploratory case history (Thomson, 2007) for each participant, building a “thick” description (Geertz, 1973) of each participant’s experiences and identifying any gradual shifts in their evolving beliefs, expectations, intentions, and experiences related to romantic relationships (subsequently referred to as “relationship trajectories”). We incorporated into each memo any complementary information from other data sources (baseline and endline questionnaires, focus groups with teachers and health educators, and field notes) to help understand and contextualize each participant’s personal or relationship trajectory. To do so, we adopted a “complementarity” approach to triangulation, which

seeks to produce a fuller picture of the research questions by combining information from different methods or different observers . . . The results are not expected to be the same, but rather to make sense in relation to each other and to help create a fuller picture of the research problem by creating more complete information about a topic. (Nightingale, 2009, p. 490)

We then conducted a thematic analysis of interview excerpts from the nine participants regarding their experiences of participating in repeat interviews, and engaged in discussions with the research team to reflect on our own experience with and perceptions of the repeat interview process. We also reviewed the case histories, analytic memos, and field notes written earlier in the process with a focus on identifying the benefits and challenges of qualitative longitudinal methods as an evaluation approach for social change interventions. We incorporated these observations into analytic memos (Saldaña, 2009).

We present four case histories that represent common types of relationship trajectories we observed and illustrate the main ways in which repeat interviews elucidated different types of information. The excerpts presented here represent typical examples that applied across the full data set. Each of the case studies represents a participant who had completed four interviews. We also describe participant experiences with the repeat interviews, drawing from all nine participants. All authors worked with quotations in the original Spanish and only translated them into English for presentation here.

## Results

We found that repeat interviews conducted during and after a social change program helped us learn about intervention effects and explore pathways to change in three main ways: first, by providing rich contextual information that helped interpret participant narratives; second, by elucidating gradual shifts in attitudes, beliefs, and behaviors among participants; third, by identifying aspects of the intervention that appear to influence change. We present four cases to illustrate each of these three uses of a longitudinal qualitative evaluation approach (summarized in Table 2).

**Table 2.** Areas of Inquiry Illuminated by Use of Repeat Interviews, by Case With Examples.

Area of Inquiry	Case 1	Case 2	Case 3	Case 4
Context and prior situation	Described family views about IPV that appear to influence his beliefs coming into the intervention	Described her parents’ relationship and its influence on what she thinks of as “normal” behavior in relationships	Provided information about his beliefs at different points in time to help explore evolving and apparently inconsistent narratives	Described observing IPV at home and strongly rejecting this form of violence
Gradual shifts in knowledge, beliefs, and behaviors	Shifts in his terminology around and definitions of IPV	Shifts in how she defined IPV and what she described as appropriate behavior in relationships	Shifts in how he described his beliefs about jealousy	Shifts in how she described her relationship and communicated with her partner about it
Influence of the intervention	Described how he put what he learned in course into practice	Said the course helped her rethink her understanding of acceptable behavior in relationships and definitions of IPV	Said the jealousy-related information in the course was important and influenced his beliefs	Said the IPV activities in the course helped her communicate assertively in the relationship and construct healthier dynamics

*Note.* IPV = intimate partner violence.

### Case 1 (Young Man, 15 Years Old)

#### Context and prior situation

In the first interview, this participant talked about his parents’ negative views of IPV. For example, his father told him never to hit a woman and his mother helped a friend leave a violent relationship. He told us he was proud that his past relationships were not violent:

I’ve never had, like, these modern relationships you could say, where there is violence, where if they don’t hit you it’s because they don’t love you, and things like that. So, I feel like, proud to say that all my relationships have been like that . . . um, healthy.

In this interview he already spoke about jealousy and controlling behavior as something to be avoided. For example, he said that “if you start to ask to check your partner’s phone, it’s because there is no trust. And so that relationship will never survive.”

#### Gradual shifts

He already spoke about avoiding jealousy in the first two interviews. For example, in the second interview, he said,

I do not like being jealous because I think that trusting your partner is essential. So, I . . . if I see there is something going on that I don’t like, instead of being jealous I try to talk things over.

From the third interview onward, he started referring to monitoring of cell phones or social media as not only something negative but also as violence. “It’s like bullying a person, looking in their personal things. I do think this is a form of violence.”

#### Influence of the intervention

He said the course had helped him realize he did not need to agree to certain demands from a partner, such as “not to accept that they want to check my phone constantly for no reason, or not to accept that they hit me, or things like that.” He also said that, based on what he learned in the course, he told a friend that she should not allow her boyfriend to monitor her phone:

Her boyfriend wanted to check her cellphone, and well, I told her that I had just remembered the topic [in the course] and . . . he didn’t have a reason to check her phone. Because he is not even her parent to be able to do that.

#### Summary

Case 1 illustrates how repeat interviews can provide contextual information about participants’ lives—in this case, regarding family member beliefs that may influence the participant; help recognize gradual shifts in how participants describe their beliefs—in this case, about controlling and possessive behaviors as forms of violence; and identify messages from the intervention that seem to influence these shifts.

### Case 2 (Young Woman, 14 Years Old)

#### Context and prior situation

In the first interview, this participant told us that trust was an important aspect of a relationship and that she disapproved of IPV, which she said included physical and psychological violence, yelling, or being controlling. In this same interview, she mentioned that her parents had a “very normal” relationship because they did not hit each other. “Between them it is good . . . They only scream at each other, it is not that they hit each other or stuff like that.” In the second interview, she told us that her father acted possessive and controlling over her mother, for example, monitoring her phone and forbidding her from doing many things—but she did not specify what she thought about this behavior.

#### Gradual shifts

She initially characterized her parent’s relationship—in which they screamed at each other and her father controlled her mother’s actions—as “normal.” In the final interview, for the first time in our conversations, she referred to jealousy and controlling behavior as potential forms of violence—“if it gets to an obsessive point.” She also talked about her own feelings of jealousy in her romantic relationships, saying she did not want to act on that jealousy by being controlling over a partner:

Well yes, I am a jealous person. But I can tell you that I am not one of those people who would tell you, “You made me jealous, and so I am not going to let you do that, and I am going to forbid you from seeing that other person.”

#### Influence of the intervention

In the second interview, this participant told us that the course taught her about IPV, how it manifests, and types of “good” or “bad” behavior in relationships. She said she learned that IPV “can start with jealousy and end with murder.” In the third interview, she said the course helped her learn how one should behave in relationships. She told us, “before . . . I didn’t pay attention to that [acceptable behavior] . . . But now that I see what’s ok and what’s not . . . I’m beginning to take that more seriously.”

#### Summary

Case 2 shows how repeat interviews can provide information about family context that helps interpret participant narratives—in this case, about the relationship between the participant’s parents; help recognize subtle shifts in how participants describe appropriate behavior in relationships; and provide insight to the influence of an intervention—in this case, how the course seems to have encouraged the participant to rethink her understandings of IPV and shift her intentions for her own relationships.

### Case 3 (Young Man, 15 Years Old)

#### Context and prior situation

In the first interview, this participant told us that his first girlfriend had been jealous and controlling, but he had not realized it was a problem at the time. He talked about different levels of jealousy, saying “Yes, obviously, as a human, yes, yes I have felt jealousy. But it did not go further . . . Never a single blow, or breakup, or shouting, or anything. There is jealousy and there is *jealousy*.” In the second interview, he spoke again about this past relationship, saying that at the time he had not known what love should be like.

#### Gradual shifts

In the third interview, he mentioned twice that he had changed his beliefs, saying that before the course, he had thought that jealousy was a form of love, but now he perceived it negatively. For example, he said,

[Before the course] I would say, if there is jealousy there is love. Or that if they are not jealous, they don’t love you, and [things] like that. But I think that was my ideology—that you always need to have jealousy because it’s a form of protection, to know that you love someone . . . [Now I think], well, that it is bad, because if you trust your partner, why are you going to be jealous?

In the final interview, 2 months later, he again spoke of jealousy as something negative. However, this time he told us he always had thought jealousy was bad—a divergence from his narrative in prior interviews.

Interviewer: What did you think about jealousy before the course?

Participant: Well, that it is bad, because you are not sure about yourself, like, you think that the person is your property and you think that someone is going to take them away. Like no, you don’t know how to trust . . . in your partner.

Interviewer: And during the course, did your perception change with regards to jealousy, or you continue to think the same way?

Participant: No. I still think the same.

#### Influence of the intervention

In the second interview, he said the course activities helped him think differently, “because you reflect [on the types of violence] and you say, ‘ah, I had this [happen to me],’ or ‘I did that.’” In the third interview, he told us that learning about jealousy was an important aspect of the course for him.

#### Summary

Case 3 shows how repeat interviews can add context about participants’ relationship experiences and beliefs prior to the intervention; provide detailed information about participants’ evolving beliefs—in this case, about romantic jealousy; and help interpret any shifts or apparent inconsistencies in participants’ narratives about the course and its influence.

### Case 4 (Young Women, 16 Years Old)

#### Context and prior situation

In the first interview, this participant already described having strong feelings against IPV because of the partner violence she observed between her parents and among other family members. She also talked about the harms of romantic jealousy and said she had intervened in a controlling relationship at school, saying to a classmate, “he [your boyfriend] does not have rights over you, he is not your owner or anything like that.” We do not know whether this exchange took place before the course began. She also told us that what she liked least about her current relationship was “that he is very jealous.” In the second interview, she told us that her boyfriend had not always been jealous, but that things started to change a few months ago, after they had sex for the first time. Since then, he had monitored her social media and phone, questioned her about her whereabouts, stopped her from talking to other men, intimidated people who spoke with her, spied on her, and told her how to dress. She said she did not like this behavior and had told him he could not control her: “If my dad doesn’t forbid me from doing things, why would you?” She described the situation as “a fight that never ends” and said she had considered ending the relationship, but every time she tried, her boyfriend cried and convinced her to stay together. She said she did not want to have sex again, because she was concerned his controlling behavior would further escalate:

When that happened [having sex], . . . it wasn’t suddenly, like the next day, right? But yes, I don’t know, maybe a week later he was like, “I don’t want you to see you like this, I don’t want to see you with him” and things like that . . . That’s why I’m saying, for it [sex] to happen again—well, I don’t think so.

#### Gradual shifts

In the fourth and final interview, she told us her relationship had changed and that she no longer experienced harmful behaviors in her relationship. “In my personal life . . . well, I have not been exposed to those situations . . . Before, I was, but not now.” She said she was fighting less with her boyfriend, and that her priority was now school rather than the relationship. She also described the relationship as something that might not last forever. She said she told her boyfriend, “just because we are dating doesn’t mean we are going to be together forever” and that “we must also focus on things that really matter to us. That is, I am not saying the relationship doesn’t matter, but at least for me the priority is to be in school and, to do well in school.”

This participant’s teacher, when asked about any changes that she had observed in the students during the course, mentioned a young woman who had been in a controlling relationship, but had lately become more confident and involved in her schoolwork—in part because of the course. Although we cannot be certain, we believe she was referring to the participant in Case 4 based on the description and details of the situation:

She is a very committed girl, very studious . . . she has very clear goals for herself. However, she was taken by this infatuation, right? Then . . . she was very controlled by her boyfriend, very controlled. We realized that her boyfriend was violent towards her . . . But now I notice her being more confident, happier. I am talking about something subjective . . . but her appearance is that she is back to that girl from the first semester, who came [to school] with enthusiasm . . . And especially this course, I think, helped her understand many things. (Teacher)

#### Influence of the intervention

When we asked whether the course helped improve her relationship, she said it did by promoting communication, and because “[the facilitators] said that a relationship is between two people, no? That you have to have trust, that being jealous does not . . . does not benefit the relationship.” The participant said she attended all the intervention sessions and paid particular attention to the IPV activities. She told us the course helped participants understand that possessive behavior is a type of IPV and that she felt more confident about what to do if she experienced it in the future. The teacher’s narrative about the influence of the course provides a complementary source of information that aligns with the participant’s description of the influence of the course.

#### Summary

Case 4 illustrates how repeat interviews can provide detailed contextual information about participants’ relationship history; help interpret participants’ narratives about their relationship trajectories; and provide information about the effects of the course—in this case, how the course promoted self-reflection and appears to have contributed to shifts in the participant’s relationship.

### Participant Experiences With Repeat Interviews

Overall, participants reported having positive experiences with the repeat interviews in this study. At the start, not all participants were comfortable sharing their feelings and personal information. Although some shared sensitive information in the first interview—including sexual assault, suicide attempts, and family and relationship violence, others did not disclose these types of experiences until later interviews. Two young women mentioned feeling nervous or uncomfortable about the idea of sharing personal information in their first interview. As one said to the interviewer, “When you and I started to have these interviews, it was like, it was like, ‘oh, how scary, and what if she tells someone else [about what we discussed]’” (Lizbeth). Both participants said they ultimately decided to share sensitive information as they felt that the interviewer was open minded and would not judge them.

Some participants said that the interviews helped reinforce the course contents and encourage further self-reflection. For example, one young woman said she liked that the interviews made her remember what was discussed in the course. Two young men noted that although all the participants in the course were given the opportunity to reflect, the repeat interviews reinforced the core topics and may have provided additional opportunities for reflection.

For some participants, the repeat interviews allowed them to discuss topics they did not talk about with anyone else, and seemed to play a therapeutic role. One participant said that interviews were a place to unburden himself, and another said the interviews “helped me like . . . to let out what I had . . . Clear my head, to . . . let off steam and then talk to someone.” Some participants introduced topics that were troubling them—such as arguments with siblings, their sexual orientation, violence at home, or social conflicts—and discussed them at length over multiple interviews. For example, one participant brought up the preferential treatment her brother received at home in three of her four interviews, spending more time discussing this than the other topics from the interview guide. Similarly, one young man volunteered information about his sexuality in the first interview (“I’m going to tell you this, I’m bisexual”) and brought the conversation back to his sexuality in all subsequent interviews. In both these cases, the repeat interviews appeared to provide an opportunity for participants to discuss their feelings and return to topics of relevance to their lives.

## Discussion

Our analysis shows the promise of repeat interviews for understanding how social change programs work in a particular time and place. Repeat interviews helped us understand why and for whom an intervention is (or is not) effective by providing information about detailed context, evolving experiences and processes of change, and the core components of the intervention that appeared particularly salient. Our findings show how a longitudinal qualitative evaluation approach using repeat interviews is useful, acceptable, and feasible as one of a broader range of complementary methods when examining complex social change programs such as IPV prevention interventions.

Theory of change techniques and repeat interviews can be used synergistically to refine and develop program theory when evaluating social change programs. In our study, the theory of change exercise informed the design of the repeat interviews, and the data collected in these interviews ultimately contributed to a more nuanced understanding of the pathways to IPV prevention. We found that participants shifted their understandings of jealousy and possessive behavior, reconsidered their beliefs about acceptable and healthy behaviors in a relationship, and identified and managed harmful behaviors in their own relationships. Measuring such incremental attitudinal and behavioral shifts, and exploring the relationship between them, is important because, first, prevention programs sometimes lead to attitudinal or behavior shifts without attaining transformational or system-level change, and second, attitudinal change can happen without behavior change, and vice versa (Gibbs et al., 2019; Jewkes et al., 2019; McLean et al., 2019; Pierotti et al., 2018). Social change involving system-level transformations, such as shifting unequal power relations between men and women or reducing community-wide levels of IPV, may require long time frames and can be more challenging to influence—and to measure—than individual-level change. Evaluations of social change interventions often prioritize measuring “final” outcomes, such as experiences of IPV—even if these outcomes may be difficult or impossible to detect within the usually short time frames available for evaluation. Because of this, in some circumstances it may be expedient to identify gradual or subtle processes of change, rather than investing in measurement of transformations that may not emerge within research time frames. This is particularly relevant for prevention interventions. Instead of waiting to see whether the behavior we seek to prevent appears over time, a longitudinal qualitative approach can provide evidence for the emergence of protective factors that come into play along the pathways to prevention.

We found that a qualitative longitudinal evaluation approach in social change interventions helped illuminate the process of critical reflection, which plays an important role in IPV prevention programming (Jewkes et al., 2019). Our results show that participants engaged in self-reflection about their past and present relationships in light of the information shared in the course, and appeared to critically examine the concepts of jealousy and controlling behavior and whether those were forms of violence. As seen in Cases 3 and 4, critical self-reflection seemed to influence participants’ relationship-related narratives, stated intentions, and to some extent beliefs.

Although critical reflection often began in the classroom—such as in response to course activities—our findings suggest it then continued during the interviews, which to some degree became an extension of the intervention. Because of this, our repeat interviews may have encouraged reflection about intervention messages beyond what would have happened through course participation alone. It is therefore possible that the repeat interview process contributed to quicker or larger shifts among our interviewees than among other participants. Other researchers have pointed out that research processes, particularly longitudinal ones, can stimulate reflection (Oakley, 2016a), be transformative for participants (Smit et al., 2021), or improve recall (Oakley, 2016b). Repeat interviews may improve our ability to detect and learn about the nature of participant experiences in an intervention due to a potentially accelerated response. In reinforcing the critical reflection component, however, they may create a false impression of what the intervention has achieved and what similar interventions might achieve without the accompanying interviews.

Repeat interviews conducted at shorter intervals may be particularly useful for data collection with young people and adolescents, who are at an age marked by rapid cognitive, physical, and psychological changes reflecting transitions to adulthood (Blum et al., 2017; Kågesten et al., 2016; Mmari et al., 2017; Joint United Nations Programme on HIV/AIDS, 2004). At this age, experiences and beliefs related to gender, sexuality, and relationships evolve quickly (Blum et al., 2017; Price et al., 2016). Many of our participants had multiple relationships during the data collection period or were experimenting sexually or questioning their sexuality (Makleff, Garduño, et al., 2020). Qualitative longitudinal methods, such as repeat interviews conducted at shorter intervals, can help capture short-duration relationships and rapid development in relationship experiences, beliefs, and identities.

Repeat interviews can provide space for developing trust and rapport between the interviewer and interviewee, which are essential for research when behaviors of interest are sensitive, stigmatized, or illegal (Calman et al., 2013; Findholt & Robrecht, 2013). Longitudinal data collection can also improve disclosure of sensitive information, as participants may become more comfortable discussing such topics in later interviews (Stern & Heise, 2019). Repeat interviews conducted frequently may be particularly useful for studies examining complex phenomena and experiences that change over time in subtle and nonlinear ways—for instance, sexuality, relationships, and violence. The interval between repeat interviews can be tailored to the lapse of time between intervention sessions, with more frequent intervention activities potentially benefiting from more frequent data collection. For example, in the context of weekly school-based intervention sessions, we found that collecting data every 1 to 2 months served to elucidate both immediate responses to recent intervention activities and the short- to medium-term effects of the IPV prevention program. It is critical to consider the outcomes being examined and frequency of intervention sessions when assessing appropriate use of a qualitative longitudinal approach in evaluation.

Qualitative longitudinal interviews can generate highly contextualized data by creating multiple opportunities for participants to share information about their lives that they find to be important. Topics raised by our interviewees were not always directly relevant to our central research questions, but they provided insight into participants’ lived experiences and their family and social contexts, and helped us understand how these may have influenced their intervention experiences. When participants repeatedly returned to certain topics over time, this provided insight to the pressing issues in their lives. The interviews may have also played a therapeutic role for some participants, a phenomenon noted in other longitudinal qualitative research (Peel et al., 2006). Providing space for participants to lead the conversations to what they care about may help mitigate power imbalances between the interviewer and the interviewee (Collins, 1998; Oakley, 2016a; Vincent, 2013).

Analyzing case histories from repeat interview data (Henderson et al., 2012; Thomson, 2007) allowed us to link participants’ individual circumstances and context with their experiences in, and responses to, the intervention. This helped explain variability in how different participants experienced the intervention (Makleff, Billowitz, et al., 2020). For example, in Cases 1, 2, and 4, participants reflected on their family situation in relation to the course contents or described discussing the intervention with family members—suggesting that violence-related beliefs and experiences at home and intervention experiences have mutual influence.

Repeat interviews during and after the intervention allowed us to track shifts in how participants talked about key course topics as they went through intervention. However, these changes sometimes made interpretation more challenging. For example, the participants in Cases 1 and 2 began to refer to excessive jealousy or possessive behaviors as forms of violence in the final two interviews—these took place at the end of the intervention and 2 months later. This may suggest a shift in their beliefs about IPV that was compatible with the aims of the intervention. However, as the intervention progressed and these messages were presented and revisited, participants may have wanted to present themselves to the interviewer in ways they believed were consistent with the course messages and, therefore, more desirable. In other words, it is important to be critical in interpreting the data, given that genuine shifts in beliefs or behaviors (what the intervention hopes to achieve) may look, at the interview, very similar to changes in how the less naïve participant wishes to present themselves in the interview—which may or may not be accompanied by deeper changes in attitude. In this study, we used a “complementary” approach to triangulation (Nightingale, 2009) when interpretation of the findings was challenging, drawing on different data collection sources and time points to help understand and contextualize the repeat interview data– for example, when narratives may have been influenced by social desirability bias.

As our findings demonstrate, inconsistencies in narratives often arise in repeat interviews, bringing challenges in interpretation. For example, the participant in Case 3 said the course helped him realize that jealousy in a relationship was “bad,” but 2 months later said he had always thought that jealousy was bad, even before the intervention. It is difficult to interpret this apparent inconsistency. Perhaps with the passing of time, the participant assumed he had always thought of jealousy in negative terms, or maybe—because excessive romantic jealousy was in conflict with the lessons of the intervention, he did not want to acknowledge that he had ever thought otherwise (or both). This raises questions of how to reconcile divergent or inconsistent narratives that may well emerge over time during longitudinal data collection. Other qualitative longitudinal studies have similarly described challenges interpreting data when participants change their narratives about a particular topic (Calman et al., 2013), reinterpret prior interactions or occurrences (Lewis, 2007), or appear unaware that their perspectives may have shifted (Grossoehme & Lipstein, 2016). Oakley suggests that such “discrepancies” between time points relate to participants having time to reflect on and process their experiences (Oakley, 2016b). Although inconsistent narratives may be common in qualitative longitudinal research, repeat interviews may provide some advantages when it comes to interpreting these, by allowing for tailored follow-up to aid in interpretation of data (Burke et al., 2019; Coventry et al., 2019; Vincent, 2013). Our strategy of reviewing transcripts and audio recordings between interviews and suggesting follow-up questions and prompts for the next interview helped us clarify narratives and explore emerging themes. Every additional interview provided scope to follow-up on emerging themes and explore apparent contradictions, which were sometimes not obvious during the interview itself.

This study has limitations. First, we were unable to begin the repeat interview process before the intervention began because of delays related to flooding and earthquakes. Because of this, we missed the opportunity to conduct “baseline” interviews prior to the interventions starting. However, the first interviews preceded the sessions about IPV—the focus of the evaluation—which took place toward the end of the intervention. In addition, we designed the interview guides to generate narratives that would allow us to explore shifts that might have been influenced by the intervention. Second, participants may have chosen to overstate their positive responses to the intervention because the interviewer worked for the organization implementing the program. Third, this analysis examined individual changes but did not explore broader shifts in social norms (e.g., changes in families or in the wider context). Fourth, our findings highlight suggestive pathways to IPV prevention, but we cannot know whether these changes will ultimately prevent or reduce IPV, or whether any changes would be sustained over time. Fifth, the repeat interview participants may differ from others in the study in terms of their willingness to share sensitive information from early on in the research process. Finally, although we included a substantial number of interviews in this analysis, the number of participants is somewhat small. The added benefits of increasing the number of participants, however, might not offset the attendant costs in terms of the labor and resources required for logistics, transcription, and analysis. We gave careful consideration to the frequency of interviews and number of participants to keep a manageable amount of data while also gathering data at intervals suitable to learn about gradual shifts over time during the intervention. In addition, the repeat interviews were not intended to stand alone—they were complemented by a range of data collection methods and sources to jointly build our understanding of the intervention.

## Conclusion

Social change programs, such as IPV prevention interventions, aim to influence attitudes, beliefs, and social norms—which can be challenging to quantify. We found that using repeat interviews as an evaluation strategy provided contextualized information about how a school-based IPV prevention program influenced participants in a particular setting. The longitudinal qualitative information we gathered helped us learn about the nonlinear and gradual pathways to IPV prevention, which can be difficult to identify with less frequent data collection. We found that repeat interviews provided meaningful empirical evidence of how the intervention influenced participants’ relationships trajectories and pathways to IPV prevention, which can be measured in the short to medium term. We conclude that a qualitative longitudinal approach, such as repeat interviews, provides a way to measure subtle changes that can serve as proxies for longer term impacts where change occurs gradually or over long periods, or if measurement is very difficult. This method can be used alongside other complementary evaluation approaches that are designed to engage a larger number of participants to build a more complete picture of intervention mechanisms and effects over time.
